# Trans‐repression of NFκB pathway mediated by PPARγ improves vascular endothelium insulin resistance

**DOI:** 10.1111/jcmm.13913

**Published:** 2018-11-05

**Authors:** Ying Kong, Yan Gao, Dongyi Lan, Ying Zhang, Rixin Zhan, Meiqi Liu, Zhouan Zhu, Guohua Zeng, Qiren Huang

**Affiliations:** ^1^ Key Provincial Laboratory of Basic Pharmacology Nanchang University Nanchang Jiangxi China; ^2^ Department of Pharmacology School of Pharmacy Nanchang University Nanchang Jiangxi China; ^3^ Jiangxi Medical College Nanchang University Nanchang Jiangxi China

**Keywords:** endothelium, inflammation, insulin resistance, NFκB, PPARγ

## Abstract

Previous study has shown that thiazolidinediones (TZDs) improved endothelium insulin resistance (IR) induced by high glucose concentration (HG)/hyperglycaemia through a PPARγ‐dependent‐NFκB trans‐repression mechanism. However, it is unclear, whether changes in PPARγ expression affect the endothelium IR and what the underlying mechanism is. In the present study, we aimed to address this issue. HG‐treated human umbilical vascular endothelial cells (HUVEC) were transfected by either PPARγ‐overexpressing (Ad‐PPARγ) or PPARγ‐shRNA‐containing (Ad‐PPARγ‐shRNA) adenoviral vectors. Likewise, the rats fed by high‐fat diet (HFD) were infected by intravenous administration of Ad‐PPARγ or Ad‐PPARγ‐shRNA. The levels of nitric oxide (NO), endothelin‐1 (ET‐1) and cytokines (TNFα, IL‐6, sICAM‐1 and sVCAM‐1) and the expression levels of PPARγ, eNOS, AKT, p‐AKT, IKKα/β and p‐IKKα/β and IκBα were examined; and the interaction between PPARγ and NFκB‐P65 as well as vascular function were evaluated. Our present results showed that overexpression of PPARγ notably increased the levels of NO, eNOS, p‐AKT and IκBα as well as the interaction of PPARγ and NFκB‐P65, and decreased the levels of ET‐1, p‐IKKα/β, TNFα, IL‐6, sICAM‐1 and sVCAM‐1. In contrast, down‐expression of PPARγ displayed the opposite effects. The results demonstrate that the overexpression of PPARγ improves while the down‐expression worsens the endothelium IR via a PPARγ‐mediated NFκB trans‐repression dependent manner. The findings suggest PPARγ is a potential therapeutic target for diabetic vascular complications.

## 1 INTRODUCTION

Insulin resistance (IR) refers to a decrease in sensitivity to insulin for insulin target tissues including muscle, liver and adipose tissue, etc and is well‐characterized as an independent risk factor for the development of type 2 diabetes mellitus (T2DM) and atherosclerosis (AS).^1^, ^2^, ^3^ Moreover, IR is also associated with a wide range of traditional and non‐traditional risk factors for cardiovascular diseases (eg endothelial dysfunction, dyslipidemia, inflammation, vascular wall abnormalities).^4^ Previously, insulin actions on muscle, liver and adipose tissue have been fully described.^5^, ^6^, ^7^ However, the role of IR in non‐canonical tissues, such as the endothelium, is less clear. Several studies support a role for IR in the development of premature cardiovascular AS independent of T2DM and obesity,^8^, ^9^ but the cause of IR is still poorly understood.

Peroxisome proliferator‐activated receptor γ (PPARγ), a super‐family member of nuclear transcription factors, plays essential roles in gluco‐lipid homoeostasis and adipogenesis and it is a molecular target of insulin‐sensitizing drugs such as thiazolidinediones (TZDs).^10^ As exogenous agonists of PPARγ, TZDs affect multiple pathophysiological processes such as lipid metabolism, vascular modifications and production of inflammatory mediators, which are involved in the development of diabetic cardiovascular complications.^11^, ^12^, ^13^ For example, TZDs decrease plasminogen activator‐1 and C‐reactive protein levels^14^ as well as reduce coronary hyperplasia after coronary stent implantation.^15^ Insulin‐sensitizing therapy with TZDs is considered as a promising intervention for patients with T2DM.^16^ However, numerous clinical studies have shown that TZDs have many side effects including fluid retention, worsening heart failure and weight gain.^17^ Therefore, reduction in side effects of TZDs especially negative effects on cardiovascular system is needed.

Although TZDs have anti‐diabetic and anti‐atherogenic effects by acting on macrophages and lymphocytes by trans‐repressing nuclear factor kappaB‐ (NFκB‐) dependent target genes,^18^ the effects of TZDs on vascular endothelial cells and the underlying mechanisms remain poorly understood. Our recent data revealed that TZDs improved vascular endothelium IR induced by high concentration of glucose /hyperglycaemia (HG) through a PPARγ‐dependent NFκB trans‐repression mechanism.^19^ However, it is unclear whether changes in PPARγ expression affect endothelium IR and what the underlying mechanism is. Therefore, in the present study, we sought to investigate the effects of alterations of PPARγ expression on endothelium IR and explore its underlying molecular mechanisms.

## 2 MATERIALS AND METHODS

### 2.1 Reagents

Dulbecco's modified Eagle's medium (DMEM) and foetal bovine serum (FBS) were purchased from Gibco‐BRL (NY, USA). The assay kits for tumour necrosis factor alpha (TNFα), interleukin‐6 (IL‐6), soluble intercellular adhesion molecule‐1 (sICAM‐1), soluble vascular cellular adhesion molecule‐1 (sVCAM‐1) and endothelin‐1 (ET‐1) were bought from R&D Systems, Inc. (MN, USA), and NO assay kit from Beyotime Institute of Biotech (Shanghai, CHN). Streptozotocin (STZ), acetylcholine (Ach), sodium nitroprusside (SNP), phenylephrine (PE) and dimethyl sulfoxide (DMSO) and all other chemicals were commercially obtained from Sigma‐Aldrich (St. Louis, MO, USA) unless indicated elsewhere. Antibodies against PPARγ, endothelial nitric oxide synthase (eNOS), AKT and phosphor‐AKT (p‐AKT), inhibitory κB kinase alpha/beta (IKKα/β) and phosphorylated‐inhibitory κB kinase alpha/beta (p‐IKKα/β) and inhibitory κB alpha (IκBα) were purchased from Cell Signaling Technology, Inc. (MA, USA), and antibody against β‐actin from Santa Cruz (CA, USA).

### 2.2 Cell lines and adenoviral vectors

The human umbilical vascular endothelial cells (HUVEC, Catalog No: CRL‐1730), 3T3‐L1 (Catalog No: CL‐173) and HEK293T (Catalog No: CRL‐3216) cells were all obtained from the American Type Culture Collection (ATCC, US). PPARγ‐overexpressing adenoviral vector (Ad‐PPARγ) and PPARγ‐short hair RND (shRNA)‐ containing adenoviral vector (Ad‐PPARγ‐shRNA) were purchased from Genechem. Tech. Inc. (Shanghai, CHN).

### 2.3 Cell culture

Cultures of HUVEC and HEK293T were performed as described in our previous study.^20^ Briefly, the cells were cultured in gelatin‐coated six‐well plates and propagated in DMEM, which was supplemented with 10% FBS, 100 IU mL^−1^ penicillin and 0.1 mg mL^−1^ streptomycin. The cells were cultured at 37°C in a 95% O_2_‐5% CO_2_ humidified atmosphere.

The culture and differentiation of the 3T3‐L1 cells were carried out as previously described in our study.^21^ Briefly, 60% confluent 3T3‐L1 cells were incubated in the serum‐free DMEM/F12 medium supplemented with an adipogenic cocktail which contains 1 m mol L^−1^ dexamethasone, 66 n mol L^−1^ insulin, 15 m mol L^−1^ HEPES, 1 n mol L^−1^ T3, 33 m mol L^−1^ biotin, 17 m mol L^−1^ pantothenate, 10 mg mL^−1^ transferrin, 100 mg mL^−1^ penicillin‐streptomycin and 1 mg mL^−1^ rosiglitazone for 8 days. During the differentiation, the medium was replaced every 2 days.

### 2.4 Establishment of endothelium IR model in vitro and in vivo

The methods for establishment of endothelium IR model in vitro and in vivo have previously been described in details.^19^ Briefly, HUVEC with a 90% confluence was first pre‐treated with a complete DMEM containing 33 m mol L^−1^ of glucose (HG) for 48 hours. After that, the DMEM was replaced by a fresh serum‐free medium and then the HUVEC was further cultured for 4 hours. Subsequently, the cells were treated with 5 mIU L^−1^ insulin (final concentration) for 10 minutes. Finally, the supernatants were collected and the levels of nitrite and ET‐1 were assayed; and the cells were used to detect the expression of AKT and p‐AKT.

All animal procedures were approved by the Institutional Animal Care and Use Committee of Nanchang University School of Medicine and conducted in accordance with the guide for the Care and Use of Laboratory Animals published by the US National Institute of Health (NIH Publication No.85‐23, revised 1996). Briefly, male Sprague–Dawley (SD) rats weighing 150‐180 g (provided by Department of Experimental Animals, Nanchang University, CHN) were fed with standard chows and water *ad libitum*, prior to the dietary manipulation. The rats were randomly allocated into two dietary regimens by feeding with either standard chows (Control, 10 rats) or high‐fat diet (HFD, 58% fat, 25% protein and 17% carbohydrate in the proportion of total kcal, 30 rats) *ad libitum* respectively, for a period of 6 weeks. At the end of 2 weeks after dietary manipulation, the HFD‐fed rats were injected intraperitoneally (i.p.) with a low dose of STZ (35 mg kg^−1^) while the control rats were given the vehicle for STZ (ie citrate buffer, pH 4.4, 1 mL kg^−1^, i.p.) respectively. Physical parameters including body weight, body length, body mass index (BMI) and fat coefficient were measured. Also, fasting plasma glucose (FPG) and serum insulin (FINS), triglyceride(TG), cholesterol (CH) as well as the homoeostatic model assay of IR (HOMA‐IR) were tested. In addition, the serum levels of nitrite and ET‐1 as well as the expression of AKT and p‐AKT from aorta tissue were assayed before modelling (pre‐model) and after modelling (post‐model).^19^

### 2.5 Expression levels of PPARγ in HEK293T and 3T3‐L1 cells with post‐transfection of different adenoviral vectors

The 90% confluent HEK293T cells were transfected with adenoviruses containing either wild‐type full‐length cDNA of PPARγ (PPARγ) or a cDNA‐scramble of PPARγ (vehicle, Veh). Similarly, 3T3‐L1 cells were transfected with adenoviruses containing either a shRNA of PPARγ (shRNA) or a shRNA‐scramble of PPARγ (vehicle, Veh). The cells that were not transfected were considered as the normal control (Ctrl). After transfection for 24 hours, fresh complete DMEM was added and these cells were further cultured for another 12 hours. Finally, the cells were harvested and PPARγ expression levels were detected by Western blots.

### 2.6 In vitro experimental protocols

The 90% confluent HUVEC was first pre‐treated with a fresh complete DMEM containing HG for 48 hours and then further cultured for 4 hours with a fresh serum‐free DMEM (IR). Next, the cells were randomly allocated to two batches. One batch of cells was transfected with adenoviruses containing either wild‐type full‐length cDNA of PPARγ (IR+PPARγ) or a cDNA‐scramble of PPARγ (vehicle, IR+Veh); while the other was done with those containing either a shRNA of PPARγ (IR+shRNA) or a shRNA‐scramble of PPARγ (vehicle, IR+Veh). The cells that were neither treated with HG nor transfected were considered as the Ctrl. After transfection for 24 hours, all the cells were washed with PBS twice and further cultured with the fresh serum‐free DMEM for an additional 12 hours. At the end, the supernatants were used to test the levels of NO, ET‐1 and cytokines (TNFα, IL‐6, sICAM‐1 and sVCAM‐1) and the cells were used to measure the expression levels of PPARγ, eNOS, AKT, p‐AKT, IKKα/β, p‐IKKα/β and IκBα. Besides, the interaction between PPARγ and NFκB‐P65 was evaluated by immunoprecipitation.

### 2.7 In vivo experimental protocols

The rats with systemic and endothelium IR were first randomly divided into five groups (Six rats per group), ie IR, IR+Ad‐PPARγ (IR+PPARγ), IR+Ad‐PPARγ‐shRNA (IR+shRNA) and their respective scrambles (IR+Veh). The rats were intravenously administered with Ad‐PPARγ (IR+PPARγ group),Ad‐PPARγ‐shRNA (IR+shRNA), their vehicles (IR+Veh groups), normal saline (IR group). The six rats that were neither treated with HFD nor transfected were considered as the Ctrl group. After treatment for a week, serum levels of NO, ET‐1 and other cytokines (TNFα, IL‐6, sICAM‐1 and sVCAM‐1) were assayed, functional assessment of rat aorta was performed, and expression levels of PPARγ, eNOS, AKT, p‐AKT, IKKα/β, p‐IKKα/β and IκBα from aorta were determined by Western blots.

### 2.8 Functional assessment of rat aorta

The functional assessment of rat aorta was carried out by a modification of the previously described method.^22^ Briefly, all the rats were fasted for 12 hours and anaesthetized with ketamine (70 mg kg^−1^, ip). Then, the rats were sacrificed by a cervical dislocation. The thoracic aorta was carefully isolated and an approximately 3 mm long aortic ring for each of the rat was prepared. The ring was isometrically mounted on a myograph (model 610M, DMT, Denmark). The aortic ring was first equilibrated for 45 minutes under a resting tension of 0.5 g and then was pre‐treated with 1 μ mol L^−1^ PE. The concentration‐response curves to acetylcholine (Ach, 10^−10^‐10^−5^ M) or sodium nitroprusside (SNP, 10^−10^‐10^−5^ M) were performed after 40%‐60% maximal contraction was induced with 1 μ mol L^−1^ PE (. The vasodilation at each concentration was measured and expressed as the percentage of force generated in response to PE.

### 2.9 Western blots for protein expression levels

Western blots were performed according to the method described in our previous study with a minor modification.^23^ Briefly, cells were washed with PBS twice, disrupted on ice for 30 minutes in NP‐40 (50 n mol L^−1^ Tris (pH 7.4), 1% NP‐40, 150 m mol L^−1^ NaCl and 40 m mol L^−1^ NaF) or RIPA lysis buffer (Thermo Scientific, Shanghai, CHN) supplemented with the protease and phosphatase inhibitors (Pierce Chemical, Dallas, US) and cleared by centrifugation. Protein concentration was determined with a BCA protein quantification kit. Equal amount of protein (35 μg) in cell lysates was first separated by SDS‐PAGE, next transferred to polyvinylidene difluoride (PVDF) membranes followed by immunoblot with specific primary antibodies at a 1:1000 dilution and second antibodies at a 1:2000 dilution, and then detected by chemiluminescence with the ECL detection reagents (Amersham Biosciences, London, UK). β‐actin was used as a loading control.

### 2.10 Quantitative assay of ET‐1 and cytokines by ELISA

Quantitative assay for ET‐1 and cytokines (TNFα, IL‐6, sICAM‐1 and sVCAM‐1) in the supernatants or in rat serum was performed using the Quantikine ELISA Kits according to the manufacturer's instruction. Briefly, 50 μL serum or cell supernatant was added to a 96‐well polystyrene microplate pre‐coated with various monoclonal antibodies against the corresponding cytokines and incubated for 2 hours at room temperature on the shaker. After five times of gentle washes with PBS, 100 μL secondary antibodies conjugated to horseradish peroxidase were added and incubated for 2 hours at room temperature, followed by an addition of 100 μL substrate solutions and incubation for 30 minutes at room temperature. Then, 100 μL stop solution was added and the optical density (OD) was read at 450 nm in a microplate reader (Bio‐Rad Laboratories, Hercules, USA).

### 2.11 Measurement of nitrite

The levels of nitrite were determined by a nitrite assay kit according to the manufacturer's instruction. Since the NO is very unstable and rapidly converted into nitrite, nitrite concentrations were measured to indicate NO levels. Nitrite reacts with Griess reagents to produce a colour, which can be measured by a spectrophotometer at 550 nm. Briefly, a standard curve was prepared using a series of nitrite concentrations. 100 μL of the sample was added into a 96‐well microplate and then the Griess reagent I and II were in turn added and mixed. Next, the mixture was incubated at 37°C for 60 minutes. Finally, the OD was determined at 550 nm with a spectrophotometer (Bio‐Rad Laboratories).

### 2.12 Statistical analysis

All data were expressed as the mean ± SEM. Significance was tested with unpaired *t* test, one‐way ANOVA and homogeneity test of variance wherever appropriate. *P* value <0.05 was considered to be statistically significant.

## 3 RESULTS

### 3.1 PPARγ expression levels in HEK293T, 3T3‐L1 and vascular endothelial cells in vitro and in vivo

**Figure 1:**
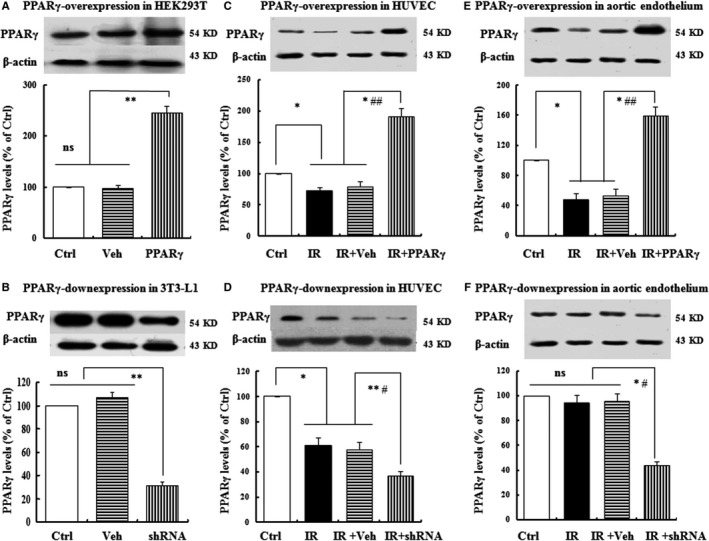
PPARγ expression in HEK293T, 3T3‐L1 and vascular endothelial cells in vitro and in vivo. The 90% confluent HEK293T cells (A) and 3T3‐L1 cells (B) were transfected with Ad‐PPARγ and Ad‐PPARγ‐shRNA respectively. Besides, the 90% confluent HUVEC were pre‐treated with freshly prepared complete DMEM containing HG for 48 h and then transfected with Ad‐PPARγ (C), Ad‐PPARγ‐shRNA (D) and their respective scrambles (Vehicle) respectively. After transfection for 24 h, the cells were washed with PBS twice and further cultured with fresh serum‐free DMEM for an additional 12 h. Finally, the cells were harvested and PPARγ expression levels were detected by Western blots. In addition, the aortal endothelia from rats tranfected with Ad‐PPARγ (E) and Ad‐PPARγ‐shRNA containing adenoviral vectors (F) were used to examine the PPARγ expression levels by Western blots. Data are expressed as mean ± SEM of 4 in vitro and 6 in vivo independent experiments, respectively. *P < 0.05, **P < 0.01, vs. Ctrl; ^#^ *P* < 0.05, ^##^ *P* < 0.01, vs. IR or IR+Veh, ns = no significance. Ctrl: normal control, IR: insulin resistance, Veh: vehicle, PPARγ: Ad‐PPARγ, shRNA: Ad‐PPARγ‐shRNA, IR+Veh: IR+vehicle, IR+PPARγ: IR+Ad‐PPARγ, IR+shRNA: IR+Ad‐PPARγ‐shRNA

To investigate the role of PPARγ in fine‐tuning vascular endothelium IR induced by HG or HFD, over‐ and down‐expressional adenoviral vectors targeting PPARγ gene were constructed. To test whether the vectors were successfully constructed, three cell lines including HEK293T, 3T3‐L1 and HUVEC as well as rat aortic vascular endothelium were used to determine the PPARγ expression levels. As anticipated, HEK293T cells had constitutive expression levels, whereas 3T3‐L1 cells had considerably abundant expression of PPARγ. Transfection of Ad‐PPARγ into HEK293T increased the expression levels of PPARγ by 248% (vs. Veh or Ctrl, Figure 1A); while transfection of Ad‐PPARγ‐shRNA into 3T3‐L1 decreased those by 29% (vs. Veh or Ctrl, Figure 1B). Moreover, constitutive PPARγ expression in the Ctrl (HUVEC in vitro and rat vascular endothelium in vivo) group was found, while that in IR or IR+Veh group decreased significantly compared with Ctrl group. However, due to transfection of the over‐ or down‐expressional adenoviral vectors, the expression levels of PPARγ were notably up‐regulated or down‐regulated respectively (vs. IR+Veh, Figure 1C,F), indicating that the vectors were successfully constructed and the transfection met the experimental needs.

### 3.2 Amelioration of vascular endothelium IR in vitro and in vivo by PPARγ

**Figure 2:**
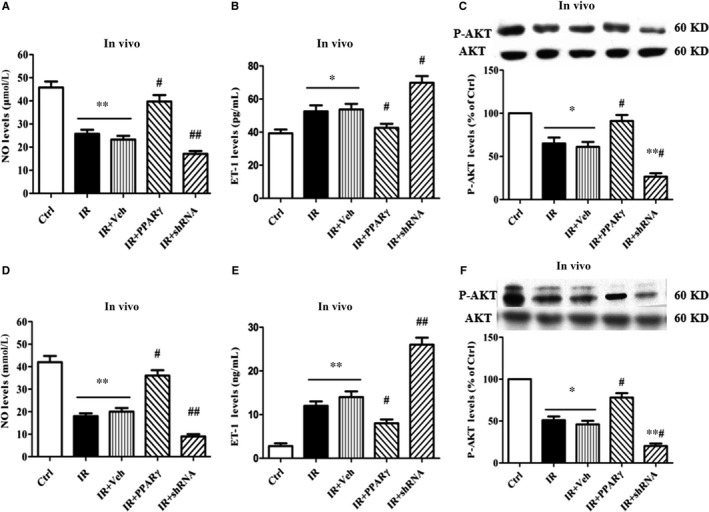
Amelioration of vascular endothelium IR in vitro and in vivo by PPARγ. The 90% confluent HUVEC were pretreated with freshly prepared complete DMEM containing HG for 48 h and then transfected with Ad‐PPARγ, Ad‐PPARγ‐shRNA and their respective scrambles (Vehicle), respectively. After transfection for 24 h, the cells were washed with PBS twice and further cultured with fresh serum‐free DMEM for additional 12 h. Subsequently, the cells were treated with insulin (5 mIU L^−1^, final concentration) for 10 min. At the end, the supernatants were collected and used for the assay of the levels of nitrite (A) and ET‐1(B); and the cells were used to detect the expression of AKT and p‐AKT (C). Besides, the serum levels of nitrite (D) and ET‐1(E) as well as the expression of AKT and p‐AKT from aorta tissue (F) in Ad‐PPARγ‐containing rats and Ad‐PPARγ‐shRNA‐containing rats were measured. Data are expressed as mean ± SEM of 4 in vitro and 6 in vivo independent experiments respectively. *P < 0.05, **P < 0.01, vs. Ctrl; #*P* < 0.05, ##*P* < 0.01, vs. IR or IR+Veh. Ctrl: normal control, IR: insulin resistance, IR+Veh: IR+vehicle, IR+PPARγ: IR+Ad‐PPARγ, IR+shRNA: IR+Ad‐PPARγ‐shRNA

The extent of endothelium IR was evaluated by the levels of NO and ET‐1 stimulated by insulin.^24^ As shown in Figure 2, HG or HFD markedly decreased the levels of NO and p‐AKT but increased the levels of ET‐1 both in vitro and in vivo (vs. Ctrl), indicating that the endothelium IR was elicited. Overexpression of PPARγ (ie IR+PPARγ group) normalized the decreased levels of NO and p‐AKT and the increased levels of ET‐1 induced by HG or HFD both in vitro and in vivo (vs. IR or IR+Veh group), demonstrating overexpression of PPARγ ameliorated considerably the endothelium IR induced by HG or HFD. However, down‐expression of PPARγ (ie IR+shRNA group) exacerbated the changes in the levels of NO and p‐AKT ae well as ET‐1 induced by HG or HFD both in vitro and in vivo (vs. IR or IR+Veh group), indicating that it deteriorated severely the endothelium IR induced by HG or HFD (Figure 2A‐F).

### 3.3 PPARγ improves endothelium‐dependent vasodilation in IR rats

**Figure 3:**
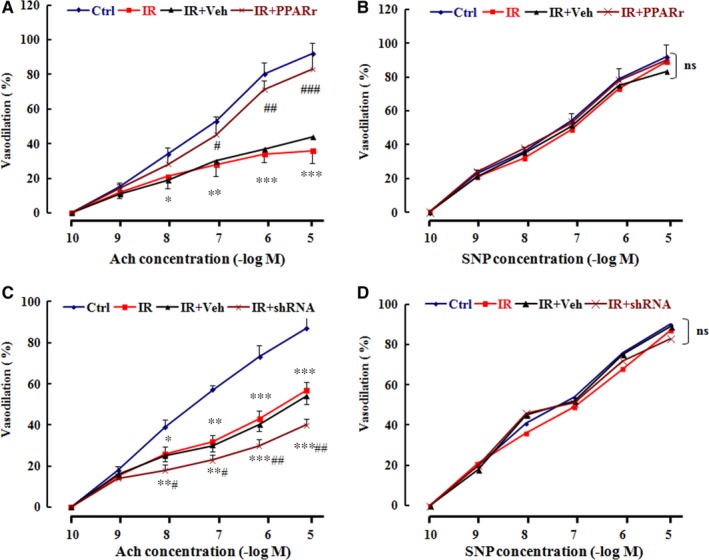
PPARγ improves endothelium‐dependent vasodilation in the IR rats The rats in IR+PPARγ or IR+shRNA group were intravenously administered with Ad‐PPARγ or Ad‐PPARγ‐shRNA respectively, while the rats in IR or IR + Veh group were intravenously given normal saline or a vehicle. The rats that were neither treated with HFD nor transfected were considered as a normal control group (Ctrl). Then, the rat aorta was used to assess the vasodilation function 1 week after treatment. Data are expressed as mean ± SEM of 6 rats. *P < 0.05, **P < 0.01, ***P < 0.001, vs. Ctrl; ^#^ *P* < 0.05, ^##^ *P* < 0.01, vs. IR or IR+Veh, ns = no significance. Ach: acetylcholine, SNP: sodium nitroprusside. Ctrl: normal control, IR: insulin resistance, IR+Veh: IR+vehicle, IR+PPARγ: IR+Ad‐PPARγ, IR+shRNA: IR+Ad‐PPARγ‐shRNA

As endothelial dysfunction occurs followed the vascular endothelial IR, we next examined the effects of PPARγ expression on endothelium‐dependent vasodilation in vivo. As expected, the endothelium‐dependent vasodilation induced by Ach in IR rats (IR group) was decreased by up to 70% compared with the Ctrl rats, whereas the endothelium‐independent vasodilation caused by SNP failed to be affected at all in IR rats (IR group). Overexpression of PPARγ, nevertheless, almost restored the endothelium‐dependent vasodilation damaged by HFD rather than the endothelium‐independent vasodilation (Figure 3A,B); down‐expression of PPARγ, in contrast, worsened the endothelium‐dependent rather than endothelium‐independent vasodilation damaged by HFD (Figure 3C,D).

### 3.4 Effects of PPARγ levels on eNOS expression in vitro and in vivo

**Figure 4:**
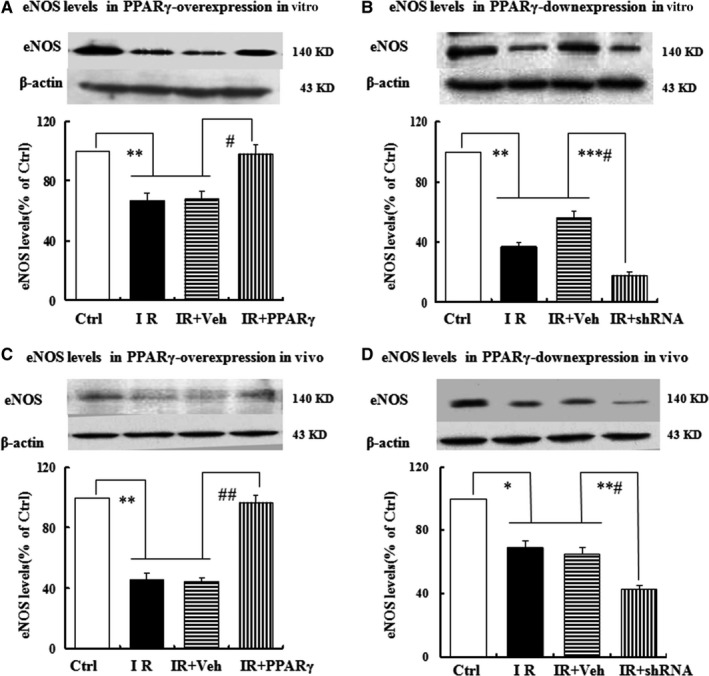
Effects of PPARγ on eNOS expression in vitro and in vivo. The expression levels of eNOS were examined by Western blots when PPARγ were over‐expressed in vitro (A) and in vivo (C), and down‐expressed in vitro (B) and in vivo (D). The grouping and treatments in vitro and in vivo were the same as described in Figures 2 and 3, and data are expressed as mean ± SEM of 4 in vitro and 6 in vivo independent experiments, respectively. *P < 0.05, **P < 0.01, ***P < 0.001, vs. Ctrl; #*P* < 0.05, ##*P* < 0.01, vs. IR or IR+Veh. Ctrl: normal control, IR: insulin resistance, IR+Veh: IR+vehicle, IR+PPARγ: IR+Ad‐PPARγ, IR+shRNA: IR+Ad‐PPARγ‐shRNA

The effects of PPARγ levels on eNOS expression in vitro and in vivo were examined. As predicted, marked decreases of eNOS expression levels were visualized both in the IR HUVEC and the IR rats. Overexpression of PPARγ, nonetheless, strikingly reversed the decreases of eNOS expression levels induced by HG or HFD and down‐expression of PPARγ expedited the decreases of eNOS expression levels both in vitro and in vivo (Figure 4A‐D).

### 3.5 Effects of PPARγ on p‐IKKα/β and IκBα expression in vivo and in vitro

**Figure 5:**
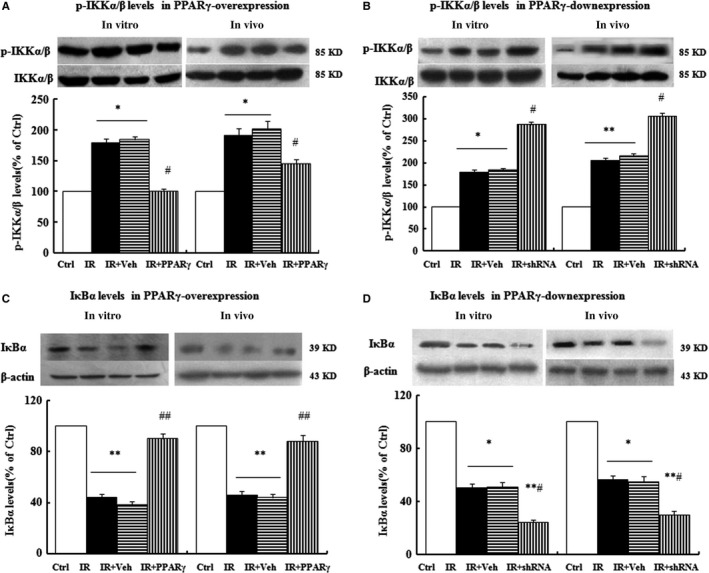
Effects of PPARγ on p‐IKKα/β and IκBα expression in vitro and in vivo. The expression levels of p‐IKKα/β and IκBα were examined by Western blots when PPARγ were over‐expressed in vitro and in vivo (A, C), and down‐expressed in vitro and in vivo (B, D). The grouping and processing in vitro and in vivo were the same as described in Figures 2 and 3, and data are expressed as mean ± SEM of 4 in vitro and 6 in vivo independent experiments, respectively. *P < 0.05, **P < 0.01, vs. Ctrl; #*P* < 0.05, ##*P* < 0.01, vs. IR or IR+Veh. Ctrl: normal control, IR: insulin resistance, IR+Veh: IR+vehicle, IR+PPARγ: IR+Ad‐PPARγ, IR+shRNA: IR+Ad‐PPARγ‐shRNA

Compared with Ctrl group, the IKKα/β level in the IR group was not significantly changed, but the p‐IKKα/β level was increased by up to 195%. However, overexpression of PPARγ dramatically antagonized the increase of p‐IKKα/β induced by HG or HFD and down‐expression of PPARγ enhanced the increase of the p‐IKKα/β level induced by HG or HFD both in vitro and in vivo (Figure 5A,B). Besides, compared with the Ctrl group, the IκBα level in the IR group was markedly decreased. Nevertheless, overexpression of PPARγ strikingly counteracted whereas the down‐expression expedited the decrease of IκBα level induced by HG or HFD (Figure 5C,D).

### 3.6 Physical interaction of PPARγ with P65 contributes to decreases of cytokines in HUVEC

**Figure 6:**
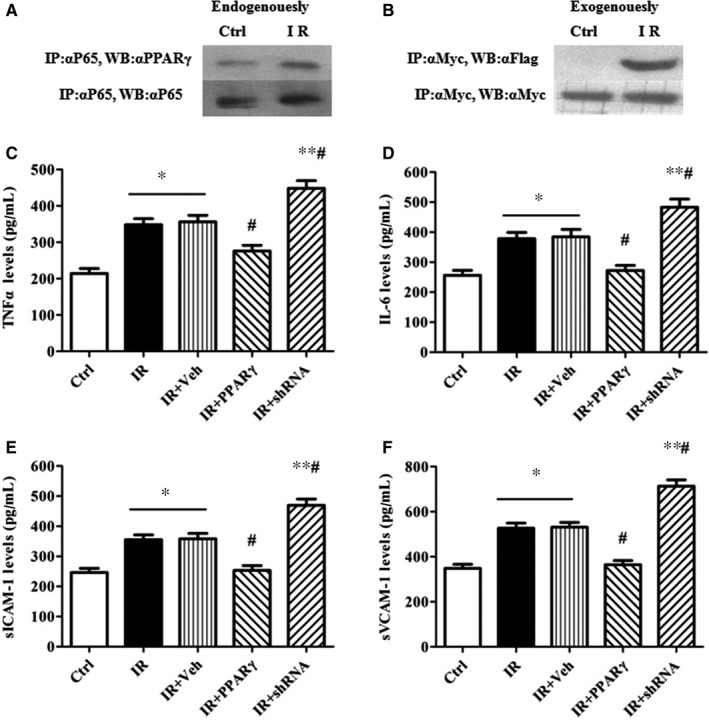
Physical interaction of PPARγ with P65 contributes to decreases of cytokines in HUVEC. The cells were harvested to assay the association of PPARγ with NFκB‐P65 by immunoprecipitation (A and B) and the supernatants were used to measure the levels of TNFα (C), IL‐6 (D), sICAM‐1(E), and sVCAM‐1(F) by ELISA. The grouping and processing were the same as described in Figure 2 and data are expressed as mean ± SEM of 4 independent experiments. *P < 0.05, **P < 0.01, vs. Ctrl; #*P* < 0.05, vs. IR or IR+Veh. Ctrl: normal control, IR: insulin resistance, IR+Veh: IR+vehicle, IR+PPARγ: IR+Ad‐PPARγ, IR+shRNA: IR+Ad‐PPARγ‐shRNA

As shown in Figure 6A,B, after exposure of HUVEC to HG for 48 hours, both endogenous and exogenous interaction of PPARγ with P65 occurred. Since the interaction of PPARγ with P65 hinders the binding of heterodimer of P65 and p50 to the promoter of target genes,the levels of cytokines including TNFα, IL‐6, sICAM‐1 and sVCAM‐1 were tested. As anticipated, the levels of TNFα, IL‐6, sICAM‐1 and sVCAM‐1 in IR group were increased by 62%, 50%, 56% and 65% respectively (vs. Ctrl). However, overexpression of PPARγ opposed whereas the down‐expression worsened the increases induced by HG (Figure 6C‐F).

### 3.7 Effects of PPARγ expression on the levels of cytokines in the IR rats

**Figure 7:**
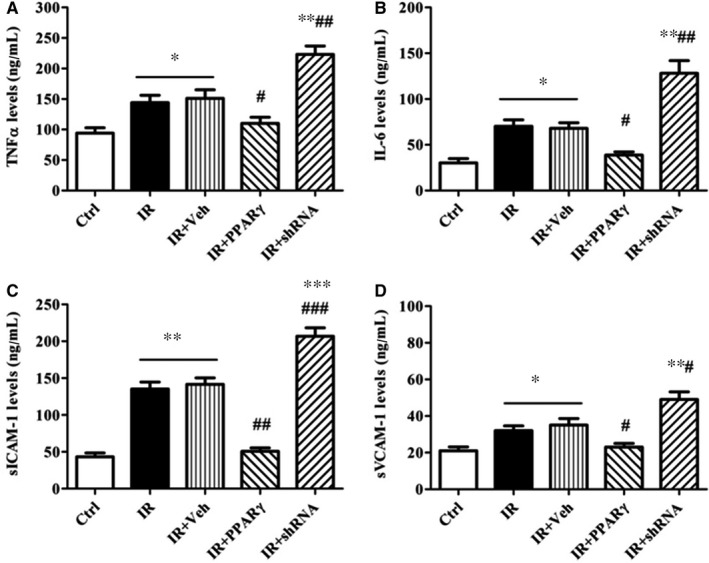
Effects of PPARγ expression levels on the levels of cytokines in IR rats. The serum levels of TNFα (A), IL‐6 (B), sICAM‐1(C), and sVCAM‐1(D) were measured by ELISA. The grouping and processing were the same as described in Figure 3 and data are expressed as mean ± SEM of 6 rats. *P < 0.05, **P < 0.01, ***P < 0.01, vs. Ctrl; #*P* < 0.05, ##*P* < 0.01, ###*P* < 0.001, vs. IR or IR+Veh. Ctrl: normal control, IR: insulin resistance, IR+Veh: IR+vehicle, IR+PPARγ: IR+Ad‐PPARγ, IR+shRNA: IR+Ad‐PPARγ‐shRNA

The levels of serum cytokines including TNFα, IL‐6, sICAM‐1 and sVCAM‐1 were tested in the IR rats. Consistent with those in vitro, the levels of TNFα, IL‐6, sICAM‐1 and sVCAM‐1 in IR rats were increased by 64%, 113%, 230% and 62% respectively (vs. Ctrl group). However, overexpression of PPARγ notably reduced while the down‐expression further elevated the increased serum cytokines induced by HFD (Figure 7A‐D).

## 4 DISCUSSION

PPARγ is a nuclear receptor that acts as a transcription factor upon activation, by regulating the transcription and expression of specific genes encoding proteins involved in insulin signalling and gluco‐lipid metabolism. PPARγ is highly expressed in adipose tissue, skeletal muscle, liver, pancreatic β‐cells, heart, colon, placenta and in the cells of vascular and immune systems^25^ and plays critical roles in regulating insulin sensitivity, gluco‐lipid metabolism and adipogenesis.^10^, ^26^, ^27^ In the current study, we have shown overexpression of PPARγ notably increased the levels of NO, eNOS, p‐AKT, IκBα and the interaction of PPARγ and NFκB‐P65, and decreased the levels of ET‐1, p‐IKKα/β, TNFα, IL‐6, sICAM‐1 and sVCAM‐1. In contrast, down‐expression of PPARγ showed opposite effects. The findings might suggest PPARγ is a potential therapeutic target for diabetic vascular complications.

In the study, we have shown that PPARγ was highly expressed in 3T3‐L1 cells, moderately expressed in HUVEC and HEK293T cells. Therefore, we chose the three cell lines to evaluate the transfection efficacy of viral vectors carrying a full‐length cDNA and shRNA targeted PPARγ gene. Both in vitro and in vivo experiments showed that the transfection of viruses carrying a full‐length cDNA of PPARγ significantly up‐regulated the expression of PPARγ, while the transfection of viruses carrying a shRNA‐PPARγ notably down‐regulated the expression of PPARγ, indicating that construction and transfection of the viral vectors were successful.

Next we tested whether the changes of PPARγ expression levels affected endothelium IR and dysfunction. It is well known that vascular endothelium is not only a vascular barrier, but also an important endocrinal organ.^28^ It secrets numerous vasoactive substances including NO and ET‐1 to fine‐tune normal vessel integrity and tension stimulated by a physiological dose of insulin.^29^ Production of NO and ET‐1 induced by insulin is modulated by IRS‐1/PI3K/AKT/NO and Raf/MAPK/ERK/ET‐1 pathways. Under normal conditions, the two pathways are kept in a balance. However, due to long‐term hyperglycaemia, hyperlipidemia and hyperinsulinemia, the balance is broken and the endothelium IR and dysfunction resultantly occurs, which results in cardio‐cerebral vessel event cascades.^30^, ^31^ Accordingly, NO and ET‐1 are often chosen as the markers of endothelium IR.^24^ In the current study, we have demonstrated that exposure of HG or HFD noticeably decreased the NO levels but increased the ET‐1 levels, resulting in the endothelium IR, and we further identified that endothelium IR and dysfunction which were induced by HG or HFD were mediated by an NFκB‐independent pathway. Over‐production of reactive oxygen species (ROS) induced by HG or HFD activated IKKα/β, a crucial component of NFκB pathway, and the activated IKKα/β interacted physically with insulin receptor substrate‐1 (IRS‐1) or Raf and further respectively phosphorylated IRS‐1 and Raf. Consequently, the insulin conventional pathway (IRS‐1/PI3K/AKT/NO pathway) was blocked, resulting in a weakening of IRS‐1/PI3K/AKT/NO pathway and an enhancement of Raf/MAPK/ERK/ET‐1 pathway (data not shown). However, overexpression of PPARγ dramatically normalized the changes of NO and ET‐1 induced by HG, while down‐expression of PPARγ expedited the changes triggered by HG. Similar results were also obtained from in vivo experiments. All of these data suggest that overexpression of PPARγ ameliorates whereas down‐expression of PPARγ worsens the endothelium IR.

As vascular endothelium IR and endothelium dysfunction appears successively in the development of AS, it is necessary to distinguish whether the changes of PPARγ expression levels affect endothelial or vascular smooth muscle function in vivo. In general, vasodilation is composed of endothelium‐dependent induced by Ach and endothelium‐independent triggered by SNP. The former reflects the endothelium function while the latter does vascular smooth muscle function.^32^ Our present data revealed that in the aorta from the IR rat, the endothelium‐dependent vasodilation induced by Ach was severely damaged but the endothelium‐independent vasodilation triggered by SNP was not at all, suggesting that in IR or early state of diabetes, dysfunction of endothelial cells occurs while the function of vascular smooth muscle cells remains intact. However, overexpression of PPARγ considerably improved the endothelium‐dependent rather than the endothelium‐independent vasodilation. In contrast, down‐expression of PPARγ significantly exacerbated the endothelium‐dependent vasodilation but had no effect on the endothelium‐independent vasodilation. Taken together, these data suggest that the changes of PPARγ expression levels indeed influence function of endothelium rather than vascular smooth muscle.

As discussed above, overexpression of PPARγ improves endothelium IR and function, which is involved in the production and availability of NO. It is well known that eNOS catalyzes L‐arginine to convert into NO in vascular endothelium under insulin stimulation. NO diffuses into VSMC where it activates guanylate cyclase (GC). Activated GC catalyzes GTP to form GMP which sequentially activates protein kinase G (PKG). PKG ultimately leads to a decrease of free cytoplasm calcium concentration, which further results in de‐phosphorylation of myosin light chain (MLC) and vasodilation.^33^ In the current study, we have shown that HG or HFD reduced the expression levels of eNOS and PPARγ. Nevertheless, overexpression of PPARγ markedly reversed the reduction of eNOS expression levels induced by HG or HFD; conversely, down‐expression of PPARγ significantly deteriorated the reduction, suggesting that eNOS‐dependent NO production is mediated by PPARγ.

Various studies have confirmed that IR and AS are a chronic inflammation process.^34^, ^35^ Besides regulating the metabolism of gluco‐lipids and adipogenisis, PPARγ has an anti‐inflammatory effect.^36^, ^37^ A large number of studies have reported that PPARγ plays above‐mentioned roles mainly via two mechanisms.^38^, ^39^, ^40^ One is the trans‐activation of PPARγ‐dependent insulin pathway, and the other is the trans‐repression of PPARγ‐dependent NFκB pathway. The former is a canonical mechanism; that is, upon activation by endogenous or synthetic ligands, PPARγ heterodimerizes with retinoid X receptor (RXR). The PPARγ/RXR heterodimer undergoes conformational changes which alter co‐activator/co‐repressor dynamics and binds further to PPRE in the promoter region of the target genes. Thus, transcription initiation of the target genes takes place.^41^, ^42^ The latter is a non‐canonical mechanism; namely, activated PPARγ/RXR heterodimer interacts physically with NFκB and impedes NFκB to bind with the promoter region of the target genes encoding inflammation factors such as TNFα, IL‐6, sICAM‐1 and sVCAM‐1 and therefore plays anti‐inflammation.^43^, ^44^

However, it is unclear whether the changes of PPARγ expression affect NFκB pathway. In the study, we have shown that HG or HFD indeed increased the levels of p‐IKKα/β and cytokines including TNFα, IL‐6, sICAM‐1 and sVCAM‐1 and decreased the IκBα levels. It might be due to the fact that activated IKKα/β, an upstream kinase of IκBα, phosphorylates IκBα at the sites of serine 32 and 36, leads to the IκBα degradation, and then gives rise to the translocation and activation of NFκB (P65/P50), and eventually causes the expression of inflammation genes (TNFα, IL‐6, sICAM‐1 and sVCAM‐1, etc). Nonetheless, overexpression of PPARγ markedly normalized the changes induced by HG or HFD and promoted the interaction between PPARγ and NFκB‐P65. By contrary, down‐expression of PPARγ significantly enhanced the changes induced by HG or HFD. These data demonstrate that overexpression of PPARγ may repress NFκB trans‐activation and improve the endothelium IR through a PPARγ‐dependent NFκB trans‐repression mechanism.

In conclusion, the present study confirmed that the changes of PPARγ expression affected endothelium IR. Overexpression of PPARγ improved endothelium IR while down‐expression of PPARγ worsened endothelium IRvia a PPARγ‐dependent NFκB trans‐repression pathway. Since loss of PPARγ function exists in the patients with T2DM and AS,^45^ the current findings suggest PPARγ is a potential therapeutic target for diabetic vascular complications.

## CONFLICT OF INTEREST

The authors confirm that there are no conflicts of interest.

## AUTHOR CONTRIBUTIONS

Y. Kong, Y. Gao, D. Lan and Y. Zhang performed the research. Q. Huang and Y. Kong designed the research. Q. Huang, R. Zhan, M. Liu, Z. Zhu, and G. Zeng analysed the data. Q. Huang and Y. Kong wrote the paper. D. Lan, Y. Zhang, R. Zhan, M. Liu, Z. Zhu, and G. Zeng reviewed and edited the paper.
